# A Case Report of Ring Avulsion Injury: Under-recognized for Its Unique Implications in Transfer

**DOI:** 10.5811/cpcem.2020.11.49917

**Published:** 2021-01-16

**Authors:** Amanda Horn, Brian Freniere, Alexander Y. Sheng

**Affiliations:** * Boston Medical Center, Department of Emergency Medicine, Boston, Massachusetts; † Lahey Hospital and Medical Center, Department of Plastic Surgery, Burlington, Massachusetts; ‡ Boston University School of Medicine, Boston, Massachusetts

**Keywords:** Ring avulsion injury, traumatic amputation, microsurgery, revascularization, digital replantation

## Abstract

**Introduction:**

Ring avulsion injuries consist of a characteristic injury pattern resulting from sudden intense force pulling on a finger ring. While ring avulsion injury is a known entity in the hand surgery literature, there is scant description of the injury pattern in emergency medicine, much less its management and transfer implications in the emergency department (ED).

**Case Report:**

This is a report of a patient presenting to the ED with ring avulsion injury after a workplace accident, initially transferred to a tertiary care hospital with general hand surgery, who then required a second transfer for consideration of microsurgical revascularization.

**Conclusion:**

In addition to fully assessing the degree of injury, including neurovascular and tendon involvement, emergency physicians must recognize cases of severe ring avulsion injuries without complete amputation as potential opportunities for microsurgical revascularization.

## INTRODUCTION

Ring avulsion injuries comprise a distinctive injury pattern that results from a sudden intense force on a ring attached to a finger. These injuries range in severity from circumferential tissue laceration to complete digital amputation.^1^ While the management of the latter is often clear-cut, treatment and disposition of ring avulsion injuries in the absence of complete amputation is more complex. As there is a paucity of emergency medicine literature with regard to ring avulsion injuries, failure of emergency physicians (EP) to recognize severe ring avulsion injuries as opportunities for microsurgical revascularization can lead to significant long-term morbidity and loss of function.^2^ In this case report, we discuss the presentation, early recognition, management, and transfer implications of ring avulsion injuries aimed at optimizing long-term functional outcomes.

## CASE REPORT

A 41-year-old-male, left-hand dominant, heavy machine operator, presented to a local community emergency department (ED) with an injury to his left ring finger after his wedding band was caught in machinery. He sustained a circumferential degloving injury to his left fourth digit, just distal to the proximal interphalangeal joint (PIP). The patient was given intravenous hydromorphone, cefazolin, and tetanus prior to transfer to a tertiary care hospital for orthopedic evaluation. The case was discussed between the transferring and accepting EPs, and disposition was deemed acceptable to both parties.

**Image 1:**
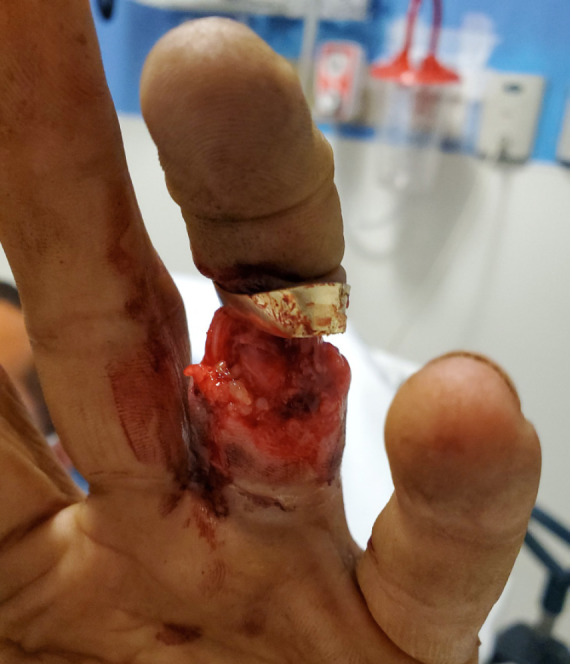
Volar view of a severe degloving ring avulsion injury.

**Image 2:**
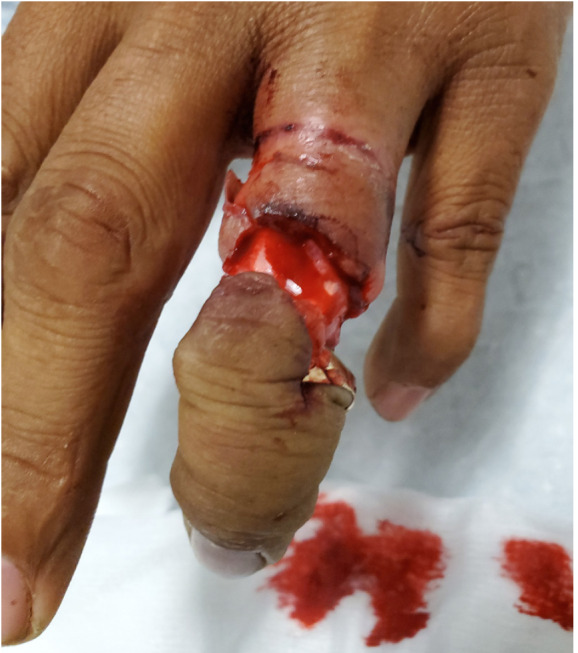
Dorsal view of a severe degloving ring avulsion injury.

Upon arrival to the tertiary care hospital ED, examination revealed a complicated ring avulsion injury with complete tendon and neurovascular disruption with exposed intact bone and distal ischemic tissue attached at the distal interphalangeal joint (DIP) (Images 1 and 2). The wedding band was deformed but still affixed to the distal soft tissue. Radiographs showed no signs of fracture. The EP contacted in-house orthopedic hand surgery. Having recognized the degree of neurovascular compromise and potential need for replantation, the EP concurrently initiated contact with hand surgery at another tertiary care hospital with microsurgical revascularization capabilities.

In a phone discussion between the EP and the in-house orthopedic hand surgeon, both parties agreed that further management should be guided by responding microsurgical revascularization specialists at the neighboring institution. After extensive discussion with the outside hand surgeon, as well as shared decision-making with the patient and family, the decision was made to transfer to a tertiary care hospital with microsurgical revascularization capabilities. Removal of the ring at this juncture was deemed unnecessary given the complete degloving of the soft tissues down to bone with complete disruption of neurovascular bundles and the clear ischemic nature of the distal soft tissues.

In the ED of the third hospital, the ring was removed after digital block to facilitate examination, revealing a central slip complete laceration, transection of both radial and ulnar neurovascular bundles, and PIP and DIP joint dislocations. The distal soft tissues were notably dusky. Given the extensive neurovascular, ligamentous, and tendon damage, as well as prolonged ischemia of distal soft tissues, there was a high likelihood of significant dysfunction even after microsurgical revascularization and aggressive rehabilitation. The decision was made with the patient and family to proceed with amputation, instead of revascularization.

**CPC-EM Capsule**

What do we already know about this clinical entity?

Ring avulsion injury consists of a characteristic injury pattern resulting from sudden intense force pulling on a finger ring.

What makes this presentation of disease reportable?

While ring avulsion injury is a known entity in the hand surgery literature, there is little to no mention of the injury pattern in emergency medicine.

What is the major learning point?

Emergency physicians must recognize cases of severe ring avulsion injuries without complete amputation as potential opportunities for microsurgical revascularization.

How might this improve emergency medicine practice?

Early recognition and transfer to microsurgical revascularization capable centers can decrease ischemic time of the distal digit and improve functional outcomes.

## DISCUSSION

While uncommon, ring avulsion injuries, when they do occur, will likely present to the ED as the first point of contact. It is crucial that EPs recognize this distinctive injury pattern and its implications for transfer, as failure to do so could result in delayed care and long-term morbidity. Initial management includes hemostasis and analgesia. The EP should ensure that tetanus vaccination is up to date and antibiotics are administered. Plain films can be obtained once the patient has been stabilized but should not delay transfer, if indicated.

A thorough assessment of the extent of damage to the soft tissues, tendons, and neurovascular structures is the critical step. Detailed examination can be facilitated by performing a digital nerve block. Further management is based on the severity and classification of injury. One such classification system based on circulatory status is the Urbaniak classification (Table). Indications to pursue repair is based on the extent of injury and individual patient priorities related to aesthetics and functionality.^3^ Lower grading class is associated with better outcomes. Class II or III injuries with questionable perfusion necessitates emergent transfer to a microsurgery-capable hand center.^4^

Amputation was previously the gold standard for class III ring avulsion injuries. However, microvascular surgery has made revascularization and replantation of higher degree injuries possible, resulting in superior functionality outcomes, measured as long-term sensibility and range of motion, compared to amputation.^5^

Discussion of these injuries should ideally be with a hand surgeon capable of microsurgical revascularization as soon as possible. Depending on injury severity, patient preferences, and local institutional resources, prompt transfer to a tertiary or quaternary care center with microsurgery capabilities must be considered.^6^ Severe ring avulsion injuries with intact bone should essentially be considered a traumatic amputation in terms of indications for transfer. When consulting a hand surgeon, it is useful to communicate the level of injury and the circulatory status of the distal digit. This may be facilitated by use of a Doppler probe or pulse oximeter.^7^ In addition, the EP should discuss whether ring removal is indicated prior to transfer as less severe injuries may still suffer from distal ischemia due to compression by a deformed ring. Factors influencing the decision to remove the ring include potential damage to surrounding structures during removal, availability of adequate ring cutter at referring institution, and possibility of delay in transfer as a result.

Although other factors are associated with survival rate of digital replantation in cases of traumatic amputations (eg, age, injury mechanism, amputated finger, length of surgery, postoperative complications, and re-intervention requirement),^8^,^9^ preserving the amputated fingers in a specimen bag filled with ice and reducing perioperative ischemic time are important elements on which EPs can exert some control. While case reports have described successful digital replantation for traumatic amputation after prolonged periods of warm ischemia,^10^ ischemic time greater than 6–12 hours is considered deleterious and expeditious door-to-surgery time of less than 180 minutes is associated with better outcomes.^11^ Given that ring avulsion injuries do not result in complete amputations, warm ischemia can quickly ensue. Thus, prompt transfer must be considered.

It remains unclear in this case whether earlier recognition for this characteristic injury pattern and its indications for potential microsurgical revascularization would have affected the eventual outcome. Nevertheless, better awareness of ring avulsion injury and its management could have prevented the additional transfer, thus decreasing the ischemic time of the distal digit.

## CONCLUSION

A thorough evaluation of ring avulsion injuries is critical to identify neurovascular and tendon injuries that require specialized care. Once this distinct injury pattern is recognized, EPs must be aware of the potential need for timely transfer to a microsurgery-capable institution rather than a general hand surgery center to optimize long-term patient outcomes.

## Figures and Tables

**Table:** Urbaniak classification for microvascular management of ring avulsion injuries.^3^

Class	Circulation	Management
I	Adequate	Treat bone and soft tissue injury
II	Inadequate	Repair vessels
III	Complete amputation or degloving	Revascularization considered; may limit functionality
